# A Detailed Algorithm for Vital Sign Monitoring of a Stationary/Non-Stationary Human through IR-UWB Radar

**DOI:** 10.3390/s17020290

**Published:** 2017-02-04

**Authors:** Faheem Khan, Sung Ho Cho

**Affiliations:** Department of Electronics and Computer Engineering, Hanyang University, 222 Wangsimini-ro, Seongdong-gu, 133-791 Seoul, Korea; faheemkhan@hanyang.ac.kr

**Keywords:** vital signs, IR UWB radar, harmonics, algorithm, respiration rate, heart rate, motion detection

## Abstract

The vital sign monitoring through Impulse Radio Ultra-Wide Band (IR-UWB) radar provides continuous assessment of a patient’s respiration and heart rates in a non-invasive manner. In this paper, IR UWB radar is used for monitoring respiration and the human heart rate. The breathing and heart rate frequencies are extracted from the signal reflected from the human body. A Kalman filter is applied to reduce the measurement noise from the vital signal. An algorithm is presented to separate the heart rate signal from the breathing harmonics. An auto-correlation based technique is applied for detecting random body movements (RBM) during the measurement process. Experiments were performed in different scenarios in order to show the validity of the algorithm. The vital signs were estimated for the signal reflected from the chest, as well as from the back side of the body in different experiments. The results from both scenarios are compared for respiration and heartbeat estimation accuracy.

## 1. Introduction

Recently, the Ultra-Wide Band (UWB) regulations have been adopted to allow unlicensed operation in the range of 3.1 and 10.6 GHz [1]. Since the legalization of UWB by the FCC in 2002, UWB technology has awakened great interest in wireless communication [2,3,4] and radar sensor applications [5,6,7,8]. UWB sensors detect the macro as well as micro movement inside the human body. The capability of non-invasive measurement of vital sign parameters of the human body is very useful in medical engineering. The deployment of an IR UWB vital measurement system may be used for the trivial vital signs monitoring of patients at home [9]. It may also be helpful for the continuous monitoring of a person while typing on a computer, driving a vehicle or sleeping on a bed. IR UWB technology has many applications due to: its robustness in a harsh environment, high precision ranging at the centimeter level, and its higher penetration capabilities [2,10,11,12,13,14]. The main reason for the usefulness of UWB sensors in medical applications is its low power consumption and large spatial resolution [11]. Non-invasive monitoring is much more appropriate in situations where it is difficult to use complicated wired connections, such as ECG monitoring for infants, burn victims or people buried during building collisions [11]. An alternative for the non-invasive detection of vital signs is microwave Doppler radar [15]. However, the Doppler systems have difficulty in penetrating materials and the null point problem [16]. The main advantage of UWB signals over the microwave Doppler radars is good material penetration. The UWB monitoring of respiration rate (RR) and heart rate (HR) has been studied in references [17,18,19,20,21,22,23,24,25,26,27,28,29] as an alternative to the Doppler-based systems [20,30,31,32,33]. The vital measurements under different circumstances such as through the wall sensing or during the motion measurement are studied by researchers [34,35,36,37,38,39]. In [11], a mathematical model for vital signals was derived and has been proved that the reflected signal from the human body contained the respiration frequency and heart rate, as well as the harmonics of these frequencies. A filter-based harmonic canceller algorithm is presented for the extraction of heart rate from the frequency transformed vital signal. However, it doesn’t provide any detail about dealing with the random body motions during the measurement process. Nguyen et al. [40] discussed the detection of motion and posture change using IR UWB radar for the monitoring of vital signs but the work lacks the effect of the posture and motion detection on vital signs. The researchers in [41] presented an auto-correlation-based algorithm that ignored the part of the signal contaminated by the motion of the body. Through the wall vital sign measurements with UWB sensors was discussed by Chai et al. [42]. A new approach based on wavelet transform is used for the estimation of body motion such as respiration motion rate [43]. In [44], an IR-UWB hardware demonstrator was presented for precise object tracking and breathing rate measurements but it didn’t contain any algorithm for the heart rate measurements. Richards et al. [45] utilized impulse radio UWB technology to alert medical personnel whenever a patient required assistance by monitoring vital signs, as well as the position of a patient inside a building. The RR and HR monitoring overnight is discussed by C. Li et al., and the measurements from four sides of the body is carried out, however, the measurements during the interval of position change was not discussed which may result in invalid measurements [46]. Yilmaz et al. [47] reviewed various wireless technologies for vital sign monitoring such as breathing, heart rate, glucose level, and blood pressure. Using UWB or Doppler radars, the breathing signal can be detected with reasonable accuracy even behind walls [20,31,32,33], which is extremely important in rescue applications. Demodulation techniques have been utilized for random body movement cancellation in quadrature Doppler radar non-invasive vital sign monitoring [48]. A noise reduction method based on improved ensemble empirical mode decomposition (EEMD), and a vital sign separation method based on continuous-wavelet transform (CWT) has been proposed to improve the signal-to-noise ratio (SNR) in order to measure RR and HR accurately [49] but the authors did not consider the strong harmonics of the breathing signal. An analytical framework for the signal-processing algorithm for vital sign measurements was presented in reference [50]; however, no method was provided to cancel the harmonics of breathing signal when the heart rate and breathing harmonics were located closely. In [51] Abdul Q. et al. detected the body motion based on the magnitude of maxima and minima in the time domain signal which might be inefficient when there is an actual change in the magnitude of the breathing signal. In [52,53] the body state of the humans is monitored using IR-UWB radar but a strategy to overcome the effect of motion on vital sign measurements was missing in these references. 

The previous work regarding vital sign measurements through IR UWB has certain limitations i.e., the effect of random body motion on the vital signs has not been studied quantitatively and an algorithm for heart rate detection in the presence of strong breathing harmonics located close to the heart rate was missing. Therefore, we studied various random body movements (RBM) to investigate its effect on vital sign measurements. The current as well as the previous spectra of the vital signal were used to estimate the heart rate and remove the strong breathing harmonics. The method can be used while sitting on a chair, driving a vehicle or lying on a bed.

**Figure 1:**
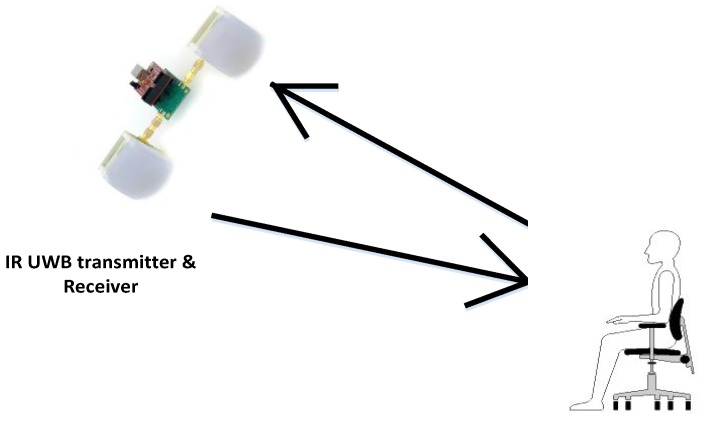
Experimental setup.

**Figure 2:**
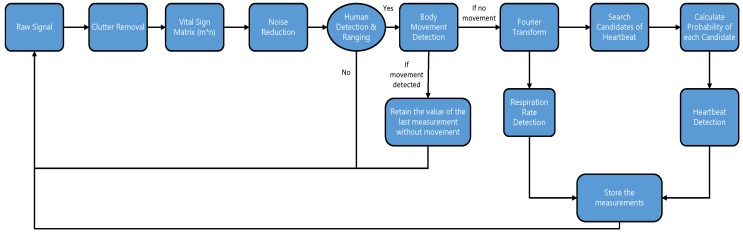
Block diagram of vital sign monitoring.

Figure 1 shows the experimental setup. The radar is connected to a computer through a USB interface and the algorithm development and signal processing has been performed using MATLAB in a Windows PC environment. The impulse radar used for the experiment was Novelda NVA6201. The hardware consisted of one RF board and one I/O module, which were connected through pin header connectors. The Sinuous Antenna was used for the experiments. The opening angle of the Sinuous antenna was from 35–40 degrees in both horizontal and vertical directions. The radiation pattern was normal to the antenna plane. The pulse repetition frequency (PRF) of the radar was 100 MHz and the center frequency of the transceiver was 6.8 GHz with a bandwidth of 2.3 GHz. The nominal output power of the transmitter was –53 dBm/MHz, which is below the FCC threshold and therefore not harmful to health. It provided a spatial resolution of four millimeters. The nanosecond pulse was achieved through a higher order Gaussian approximation impulse generator. The output center frequency and hence relative bandwidth was configurable. A part of the signal transmitted from the transmitter antenna was reflected back due to the higher reflectivity of the human body. The received signal had information relating to the environment and the human body. The signal reflected from the human body contained breathing and heart rate frequencies. The normal RR of a human being ranges from 12 to 16 times a minute and heart rate varies between 60 to 100 times per minute. However, we selected the heart frequency range from 0.8 to two hertz as the heart rate is higher in some cases such as after heavy exercise or other physical activity. The main component of the displacement of the chest is due to breathing while a small portion is due to heartbeats. Therefore, the frequency with the highest magnitude refers to the RR whereas the HR has a lower magnitude when compared to the RR. It may be easy to identify the RR as it has the largest amplitude, and the frequency range of HR doesn’t overlap the RR. However, HR might be difficult to extract when breathing harmonics have a relatively higher magnitude than the HR. A. Lazaro et al. [11] presented a notch filter-based solution to cancel the breathing harmonics; however, if the breathing harmonics occur close to the HR then it becomes very hard to filter the harmonics as it may also suppress the HR. In our work, we present an algorithm to find the heart rate based on the probability of occurrence through certain iterations. The system block diagram is given in Figure 2. 

The unwanted clutter signal needs to be removed from the raw signal. A clutter removal method based on loopback filter is employed [54]. After removing the clutter from the signal, the next step is to find the location of the human chest. We need to store each raw signal waveform and finally combine those waveforms into a matrix of size “m×n”. The ‘m’ represents the slow time length whereas the ‘n’ represents the fast time axis along each waveform. The slow time index ‘m’ can be selected by the user. The higher value of ‘m’ result in better frequency resolution but slow change in the respiration and heart rate values over time whereas if its value is chosen smaller then it may result in lower frequency resolution but fast variation in respiration and heart rate. The range of the radar sensor is configurable through parameter ‘Frame stitches’. Next, the column of interest was found in the matrix, which contained the periodic motion caused by the contraction and relaxation cycles of the lungs and heart. 

**Figure 3:**
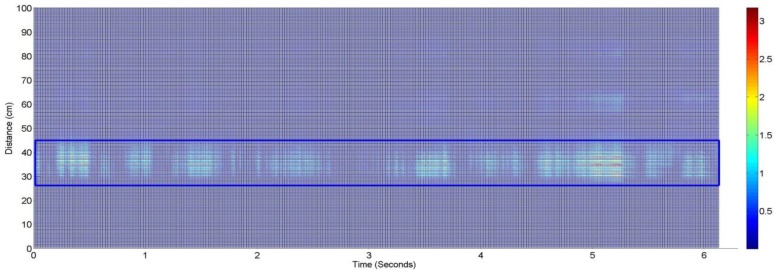
Waveforms after clutter removal where the part highlighted by the rectangle shows the reflection from the human body.

**Figure 4:**
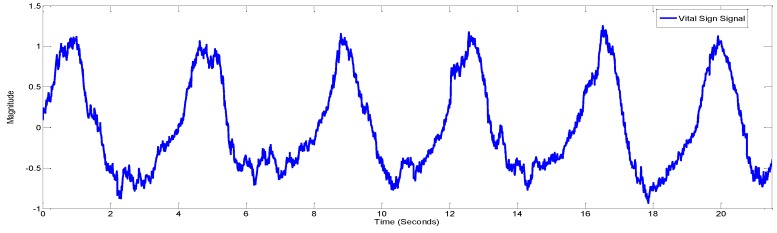
The column with highest variance/energy.

In Figure 3 the matrix of size “m×n” is shown. We found the variance of all the columns of this matrix and chose the column with the highest variance as the position along the fast time axis with highest movement [11]. The vital signal is plotted in Figure 4. 

The Kalman filter (KF) is an online recursive algorithm used to estimate the system state with noise contaminated observations [55,56,57]. In our system, the measurement noise may be reduced by applying the Kalman filter estimation to the time varying signal. 

The rest of the paper is divided as follows. In the second section, a motion detection algorithm based on the auto-correlation concept is presented. Section 3 is about the detection of respiration and heart rate and in Section 4 experimental results are presented for different scenarios and the proposed algorithm is compared with the conventional filter-based approach. In Section 5, our conclusions are presented and future work is discussed.

## 2. Motion Detection

**Figure 5:**
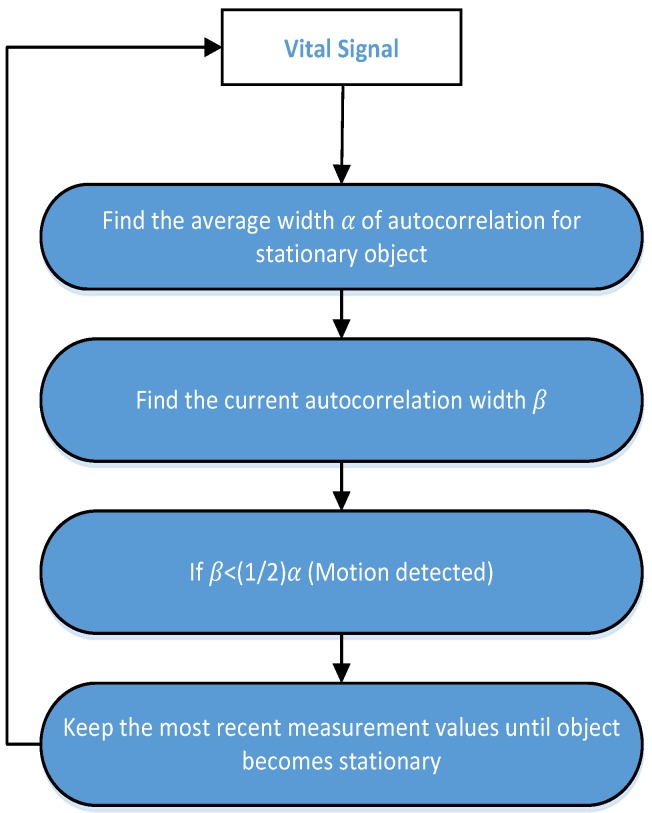
Random body motion detection algorithm.

For the experiments looking at vital signs, it was assumed that the human subject remained stationary during the measurement period but that it is unnatural to stay stationary for a long period of time, therefore, a motion detection algorithm was employed to detect the motion of the human during the measurement process. The concept of auto-correlation for motion detection is explained in Figure 5. For a stationary human, the auto-correlation width is greater than compared to the moving human, as there is comparatively less correlation among the signal samples when a person makes random body movements (RBM). If the correlation width is lower by a certain ratio, then we can determine that the person is moving and thus stop the measurement of vital signs until the person becomes stationary again. When the measurement process is stopped due to RBM, the last measurement values of RR and HR are assumed as estimated values until the RBM is finished. The implementation of the motion detection algorithm in the vital sign measurement process, the invalid values of RR and HR may be eliminated during the measurement. The detailed results on different RBM and its effect on vital signs are discussed in the experimental results section.

**Figure 6:**
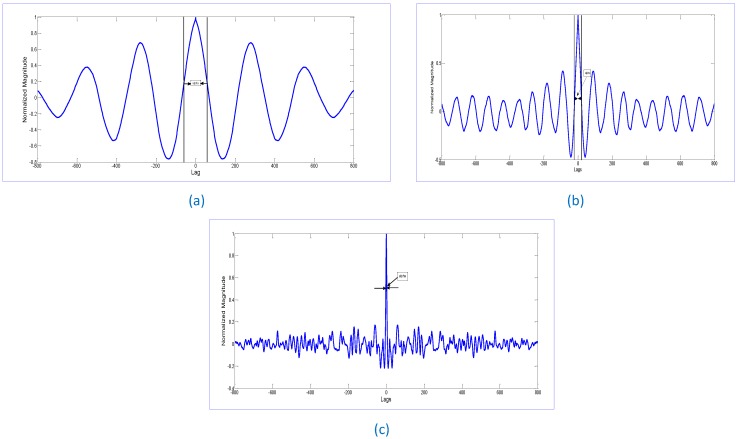
Auto-correlation signal of an object when it is: (**a**) stationary; (**b**) starts moving; and (**c**) movement detected.

In Figure 6, the concept of auto-correlation-based movement detection is explained. Figure 6a shows the width of the autocorrelation for a stationary human, Figure 6b shows the auto-correlation width when the movement has just started and finally Figure 6c is the correlation signal for the signal when the correlation width is much less and is considered as the movement detection.

## 3. Respiration and Heart Rate Measurement 

### 3.1. Frequency Domain Signal and Respiration Rate

After removing the measurement noise from the vital signal, the next step was to convert the signal from time domain to frequency domain by using Fourier Transform. 

**Figure 7:**
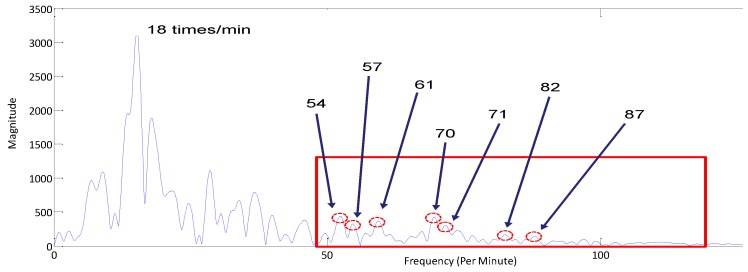
The spectrum of the vital signal.

As shown in Figure 7, the spectrum has the strongest peak value at 18 (per minute). This highest peak represents the fundamental RR. The breathing harmonics, as well as HR components are also present in the spectrum. The next goal was to search for the HR in the frequency range of HR. The authors of [11,29] proposed a notch filter bank to remove the harmonics of breathing from the signal and then search for the HR. However, if the HR occurs close to the breathing harmonics, then the filter may suppress the HR as well as the breathing harmonics. In Figure 7, the peak at 71 is at the fourth harmonic of breathing while the HR is 70 bpm, so a notch filter might cancel the HR along with the breathing harmonics. In the next section, we discuss: the algorithm, how statistically we chose HR among the locations of peak values from the current spectrum, as well as the previous signal spectra. 

### 3.2. Heart Rate Detection Algorithm

Here an algorithm is presented to extract the HR by using spectra of ‘N’ iterations and selecting the peak location based on the probability of occurrence. In previous studies, only the current spectrum was taken into account for the extraction of the HR while we have incorporated the last ‘*N*’ spectra information in our algorithm to have an accurate estimate of the HR. Our algorithm was designed under the assumption that the heart rate doesn’t change abruptly. If the heart rate changes abruptly then it might result in erroneous estimation.

**Figure 8:**
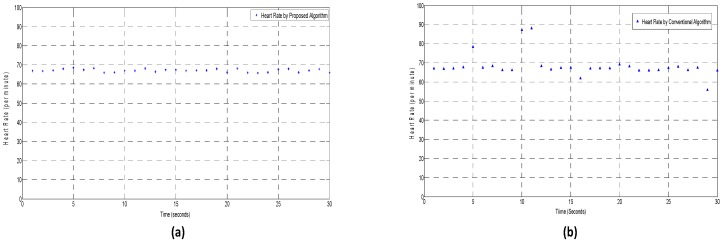
A comparison of the conventional algorithm and the proposed algorithm for heart rate monitoring: (**a**) proposed algorithm result, and (**b**) conventional algorithm result.

**Algorithm 1:** Selection of the Heart Rate1.Start the first iteration. Initialize t=1:2.From the frequency domain signal, find the highest frequency peak in the range of 10 to 30 cycles per minutes. This highest peak location value is the **RR**.3.Select the locations of the peaks in the **HR** frequency range (as shown in red colored rectangle in Figure 8). The number of peaks may vary; we chose it as ‘3’ for our experiments.4.Discard all those peaks which are at the integer multiples of **RR** i.e., discard peaks at 54, 72, 90 in Figure 85.Increment the count of each peak location when it repeats at any iteration.6.If t = N, then go to step 7: Else *t* → *t* + 1: Go to step 2.7.After the completion of ‘*N*’ rounds, check for frequency with highest occurrence.8.If one location is repeated the most then select it as **HR**, Else if two or more locations have the same occurrence then decide **HR** on the basis of average magnitude of the peaks with same repetitions.

In the above algorithm, the magnitude of the peak is not the primary criteria for HR selection, rather the number of repetition of that peak for ‘N’ iteration as the intermodulation products of the harmonics may have a higher magnitude than the heart beat rate. Therefore, we chose ‘M’ peaks during each iteration in the heart frequency range and repeated it for ‘N’ iterations. The disadvantage of this algorithm is the higher initialization time; however, once the system is initialized, a sliding window concept is used for the iterations and hence there are no further delays in the continuous non-invasive monitoring of the vital signs. The experimental results for the statistics of the Algorithm 1 are shown in Table 1 below. 

In Table 1, it is clear that ‘70’ has the highest repetition rate, i.e., 19; therefore, it is the estimated heart rate which is exactly the same as that measured through the physical sensor. 

## 4. Experimental Results

### 4.1. Noise Reduction by Kalman Filtering

**Figure 9:**
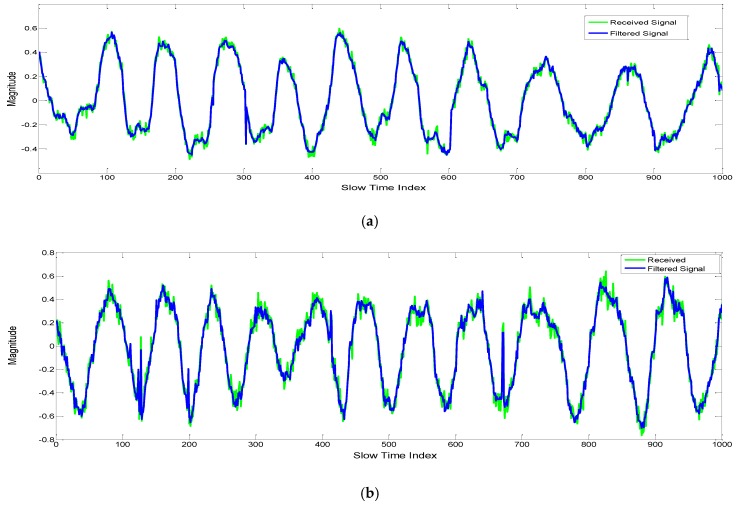
Vital signal obtained before and after Kalman filter (KF) at different locations: (**a**) at a distance of one meter from the radar, and (**b**) at a distance of two meters.

The Kalman filter is employed for reducing the error in the time domain received signal. The Kalman estimation results for the vital sign measurement of a person aged 28 years and sitting on a chair at two different ranges are shown in Figure 9. Figure 9b shows increased noise level when compared to Figure 9a, therefore it is important to reduce the noise using filtering techniques. 

The filtering becomes more important when the distance increases as shown in Table 2, the SNR is degraded with increasing distance and hence the root mean square error (RMSE) increases. By employing the Kalman filter, the RMSE for vital measurement is reduced as shown in Table 2. 

### 4.2. Heart Rate Detection by Conventional vs. Proposed Algorithm

We performed the vital sign experiments using both the conventional algorithm, as well as the proposed algorithm. The update rate was one second for both respiration and heart rate measurements. A sliding window concept was used for the vital sign matrix construction i.e., the new samples at current time replaces the previous samples. The sampling frequency for the experiment was observed to be 92.71 samples per second. The true value for HR is 67.2 per minute.

It is clear from Figure 8 that the conventional algorithm shows some invalid estimates which may be due to the fact that strong breathing harmonics and/or intermodulation components have higher values than the HR peak; whereas the proposed algorithm has much more stability and accuracy in the measurement values. 

To show the stability of the proposed algorithm over a certain period of time and prove the accuracy of the measurements, we defined the estimated values with a larger deviation from the true values as invalid estimations. The following equation defines the invalid HR estimates:

(1) if |Original Value−Estimated Value|>3

The vital signs of five different people in the age group of 23 ± 3 years were measured by using both the conventional FIR-based method, as well as the proposed algorithm. The proposed algorithm outperforms the FIR-based algorithm when the RR harmonics occurs close to the HR and have relatively higher magnitudes. Table 3 shows the invalid estimates by FIR-based algorithm and proposed algorithm. 

Table 3 clearly shows that the proposed algorithm is more stable than the conventional algorithm because the filter suppression algorithm shows some invalid estimates of heart rate when the harmonics of RR and HR are located close to each other such as for human subjects #02 and #03. 

### 4.3. Vital Signs Measurement with and without Movement Detection Algorithm

**Figure 10:**
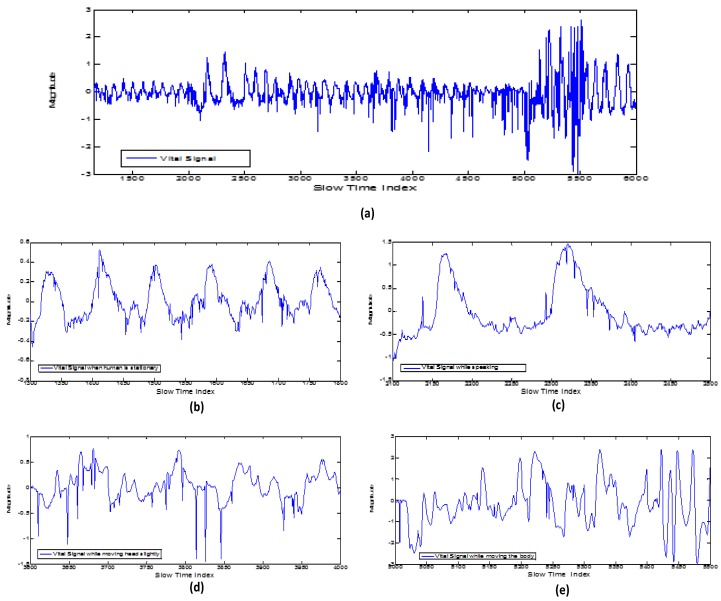
Vital signal obtained during different motion states of the body: (**a**) vital signal with different body states; (**b**) when body is stationary; (**c**) while speaking; (**d**) moving head slightly; (**e**) moving whole body slightly.

Different RBM are considered for the experiments to show its effect on vital sign measurements. The human subject is considered to be sitting in a chair facing the radar while making small RBM. Three different kinds of motions i.e., speaking, shaking head slightly and slight motion of whole body were considered. In Figure 10, the vital signals obtained during each motion period are plotted against the slow time axis:

It is concluded in Table 4 that even small RBM affects the measurement process of vital signs. Therefore, we suggest in Section 2 that the measurement process be stopped until the human becomes stationary. Table 4 shows the effect of the RBM on the vital sign measurement. 

Table 4 clearly shows that the measurement HR values are invalid in the case of speaking, head and body motion, whereas breathing rate is decreased during speaking; however, it is valid as we breathe extremely slowly during speaking. The estimated RR values during head and body motion are invalid. The time required to detect the RBM is longer for a speaking period while it is the least for body motion as it heavily distorts the signal and the auto-correlation coefficient minimizes after a single frame. 

Furthermore, we also compared the results of the estimated RR and HR with and without the motion detection algorithm. 

**Figure 11:**
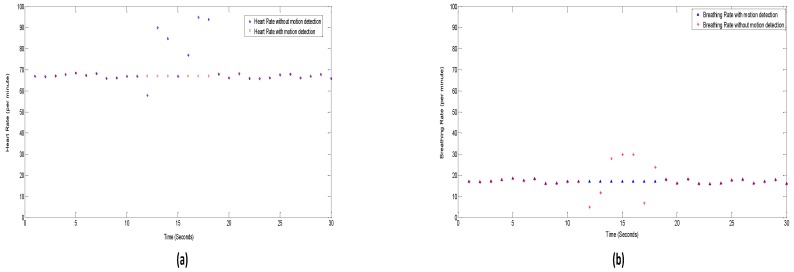
Results of vital signs: (**a**) with and (**b**) without motion detection algorithm.

Figure 11 clearly shows that the algorithm with motion detection avoids invalid estimates during the RBM. In Figure 11, the movement detection starts at around 12 s and ends at 18 s. During the RBM period, the conventional algorithm resulted in invalid estimates of RR and HR, whereas in our proposed algorithm, RBM were detected and the measurement value of the last sample (11th sample in this case) kept as the output value until the motion of body stops and the measurement process is resumed. 

### 4.4. Vital Signs Measurement with the Radar Pointed at the Backside of Body

**Figure 12:**
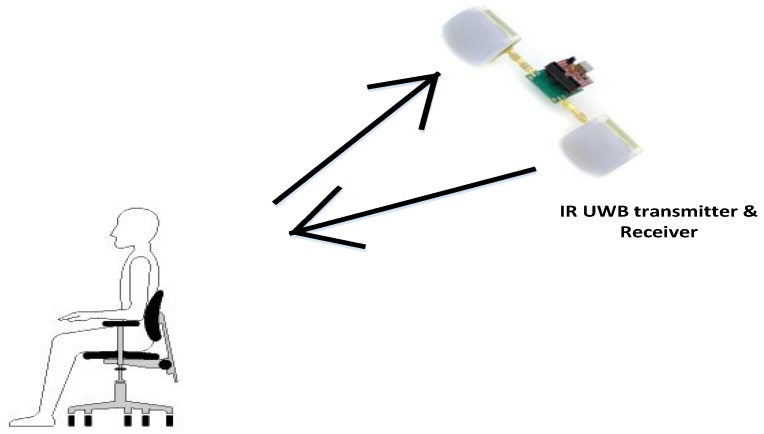
Experimental setup when the radar is pointed at the back of the subject.

The radar sensor was installed and a person was sitting with his back facing the radar as shown in Figure 12. The results showed quite good performance for heart rate while the RR was not as accurate.

After applying the detailed algorithm for vital signs, the spectrum obtained for the experimental setup in Figure 12 is shown below. 

**Figure 13:**
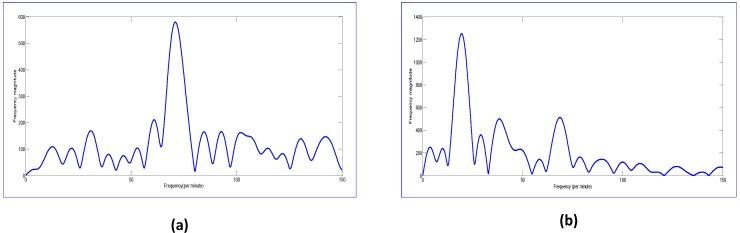
Spectrum of the signal reflected from backside of the person. (**a**) when a person is in normal relaxed mode; and (**b**) when a person is examined after heavy exercise and breathing heavily.

In Figure 13a, the reflected signal from the backside is shown. As the breathing motion is most observable from the chest part of the body, it is not very high when it is observed from the backside of the body. Therefore, the breathing signal amplitude at 20 bpm is not the highest peak as it was in the case when the signal was observed when the radar was in the front of the chest. In this case, the HR is the location of the highest peak, which occurs at 68 per minute. Figure 13b shows a spectra taken after some heavy physical activity such as exercise. In that case, the breathing magnitude is higher due to the higher expansion and contraction of the lungs and chest cavity. Therefore, the RR has the highest peak value when compared to a relaxed person. The HR is still observable and it occurs at 68.31 per minute.

## 5. Conclusions 

In this paper, we discussed the vital sign monitoring of a human through IR UWB radar. The respiration rate was chosen to be the location of the highest peak value in the signal spectrum. A Kalman filter was employed to reduce the measurement errors and the results of noise reduction for different SNR values and its performance for vital signs measurement was discussed. A statistical algorithm based on the current as well as previous spectra of the vital signal was presented for heart rate measurement and its accuracy is proven by comparing it with the conventional filter-based approach for harmonic cancellation. Moreover, for continuous non-invasive measurement of vital signs it was necessary to detect random body movements (RBM) during vital sign measurement. An auto-correlation-based method was presented for the motion detection of human body parts. The motion detection was integrated with the vital sign measurements so that the invalid measurements were avoided by cancelling the motion contaminated observations. The results proved that there are more outliers in the measured values by the conventional algorithm as compared to the values by the proposed algorithm. The respiration and heartbeat rates were measured by pointing the radar at the back side of the body which might be useful during driving situations inside a vehicle. The results for the heart rate were much better when we measure it from the back side because the respiration harmonics were not much stronger. In the future, more research is necessary on the use of motion detection algorithms with vital sign monitoring for sleep monitoring, the detection of changes in the heart rate (heart arrhythmia), and monitoring the vital signs of infants. 

## Figures and Tables

**Table 1:** The repetition of every peak frequency location.

Frequency (per Minute) Location	Total No. of Repetitions in M = 20 Iterations
57	12
62	8
70	19
82	13

**Table 2:** RMSE for vital signals with different input signal-to-noise ratio (SNR) values.

Distance b/w Human & Radar	SNR before Filtering (dB)	SNR after Filtering (dB)	RMSE Breathing Rate/Heart Rate (Unfiltered Signal)	RMSE Breathing Rate/Heart Rate (Filtered Signal)
1 m	12.3	18.2	0.012/0.840	0.006/0.372
2 m	8.6	14.9	0.042/1.831	0.029/0.487

**Table 3:** Accuracy of proposed algorithm compared to conventional algorithm for different persons.

Human Subject No.	Breathing Frequency	Heart Rate	Total Estimates	Invalid Estimates by Filter Suppression	Invalid Estimate by Proposed Algorithm
01	16	68	30	1	0
02	18	54	30	5	0
03	20	59	30	6	2
04	15	72	30	1	1
05	17	81	30	0	0

**Table 4:** Vital Signs during different motion states of the body.

Body State	Normalized Autocorrelation Width	Time Required for Motion Detection	Estimated Breathing Rate/Heart Rate	Original Breathing Rate/Heart Rate
Stationary	1	NA	13/74	13/74
Speaking	0.73	3.2 s	8/82	7.5/74
Shaking head slightly (1–3 cm)	0.46	1.8 s	19/60	13/74
Moving the body slightly (1–2 cm)	0.24	0.46 s	17/55	13/74

## Abbreviations

The following abbreviations are used in this manuscript:

IR UWB	Impulse Radio Ultra-Wideband
FCC	Federal Communications Commission
FIR	Finite Impulse Response
KF	Kalman Filter
RR	Respiration Rate
HR	Heart Rate
GHz	Giga Hertz
I/O	Input Output
RF	Radio Frequency
RBM	Random Body Movement
RMSE	Root Mean Squared Error
NA	Not Applicable
SNR	Signal to Noise ratio
Sec	Seconds
Bpm	Beats per minute
